# Statistical Significance of Earth’s Electric and Magnetic Field Variations Preceding Earthquakes in Greece and Japan Revisited

**DOI:** 10.3390/e20080561

**Published:** 2018-07-28

**Authors:** Nicholas V. Sarlis

**Affiliations:** Section of Solid State Physics and Solid Earth Physics Institute, Department of Physics, National and Kapodistrian University of Athens, Panepistimiopolis, Zografos, 157 84 Athens, Greece; nsarlis@phys.uoa.gr; Tel.: +30-210-727-6736

**Keywords:** event coincidence analysis, receiver operating characteristics, seismic electric signals, ULF seismo-magnetic phenomena, earthquake precursors

## Abstract

By analyzing the seismicity in a new time domain, termed natural time, we recently found that the change of the entropy under time reversal (*Physica A* **2018**, *506*, 625–634) and the relevant complexity measures (*Entropy* **2018**, *20*, 477) exhibit pronounced variations before the occurrence of the M8.2 earthquake in Mexico on 7 September 2017. Here, the statistical significance of precursory phenomena associated with other physical properties and in particular the anomalous variations observed in the Earth’s electric and magnetic fields before earthquakes in different regions of the world and in particular in Greece since 1980s and Japan during 2001–2010 are revisited (the latter, i.e., the magnetic field variations are alternatively termed ultra low frequency (ULF) seismo-magnetic phenomena). Along these lines we employ modern statistical tools like the event coincidence analysis and the receiver operating characteristics technique. We find that these precursory variations are far beyond chance and in addition their lead times fully agree with the experimental findings in Greece since the 1980s.

## 1. Introduction

Since the 1980s continuous monitoring of the variations of the electric field (in the frequency range ≤1 Hz) of the Earth has started in Greece. This led to the conclusion that there exist transient changes of the Earth’s electric field, termed Seismic Electric Signals (SES), preceding earthquakes (EQs) [1,2]. These changes have been classified into single SES (meaning that one transient change appears before an EQ) or SES activity which corresponds to the case when several transient changes are recorded within a short time [3]. The lead time Δt in the former case is Δt≤ 11 days (d) while in the latter case Δt is appreciably longer around a few months or so with maximum $\approx 5\frac{1}{2}$ months [4] and minimum ≈ a few weeks [3,5] (e.g., 22 d see [3]). SES are believed [5] to be generated when the gradually increasing stress in the EQ preparation area reaches a critical value $\sigma_{cr}$ and then the existing electric dipoles that have been formed due to point defects (e.g., [6]) exhibit a cooperative orientation, thus leading to the emission of a transient electric signal. This SES generation model is considered to be unique [7] among other models in that SES would be generated spontaneously during the gradual increase of stress without requiring any sudden change of stress such as micro fracturing. This model is confirmed in the following sense: Upon analysing the time series of the pulses comprising an SES activity in a new time domain, termed natural time χ, it has been shown [8,9] that these pulses exhibit infinitely ranged temporal correlations which is characteristic of criticality. For a time series comprising *N* events, we define an index for the occurrence of the *k*-th event by $\chi_{k}=k/N$, which we term natural time. In this analysis [4,10], we ignore the time intervals between consecutive events, but preserve their order and energy $Q_{k}$ because we consider that these two quantities are the most important for the evolution of the system. We, then, study the pairs $\left(\chi_{k},Q_{k}\right)$ of ($\chi_{k},p_{k})$ where $p_{k}$ is the normalized energy for the *k*-th event. In addition, in natural time analysis, all SES activities fall [4,8] on a common curve (universal) and this *universality* is also characteristic of criticality.

As for the SES transmission, the following model has been put forward [5] by considering that EQs occur near faults and in addition the electric conductivity of faults is usually orders of magnitude larger than the average conductivity of the surrounding medium (host rock), thus they constitute highly conductive paths: When the SES is emitted from the EQ preparation area, most of the current follows the highly conductive channel which may terminate below the Earth’s surface say at epicentral distances of the order of 100 km. By applying either numerical solutions of Maxwell’s equations [11] or analytical models [12], we find that the electric field *E* at a measuring site lying at the Earth’s surface, very close to the termination point is of the order of 5–10 mV/km which agrees with the experimental values (reported from various countries, e.g., Japan [13,14,15,16,17], China [18,19,20], Mexico [21,22], Kyrgyzstan [23] etc.) if we assume a current dipole source of the order of $10^{6}$ Am which corresponds to an EQ of magnitude M∼5 (e.g., [11,12]). These calculations also lead to magnetic field (*B*) values of the order $10^{-2}$ nT, which are not readily detectable. This agrees with experimental observations (e.g., [24]) that small amplitude SES (i.e., those related with EQs of magnitude M∼5) are not accompanied by easily observable variations of the horizontal components of the magnetic field on the Earth’s surface. On the other hand, strong SES activities, e.g., related with EQs of moment magnitude $M_{w}>6.0$ (hence surface wave magnitude Ms(ATH) ≥6.5, where Ms(ATH) = ML(ATH) + 0.5, and ML(ATH) is the EQ local magnitude reported by the Institute of Geodynamics of the National Observatory of Athens (ATH), GI-NOA) have been observed in Greece [25] to be accompanied by detectable magnetic field variations of the order of a few to several tens of nT mainly in the vertical component in agreement with theoretical calculations [26,27]. Such magnetic field variations are sometimes alternatively termed by some Japanese scientists ultra low frequency (ULF) seismo-magnetic phenomena, e.g., [28,29]. Since the installations for the detection of these variations are appreciably easier to construct compared to those for Earth’s electric field measurements, which require more tedious installation and analysis, they have been the object of several publications, e.g., [28,29,30,31].

An additional fact showing the physical interconnection of SES activities with seismicity has been uncovered in [32]. Concerning seismicity, we consider the view that earthquakes are critical phenomena (where a mainshock is the new phase) and the quantity by which we can identify the approach of a dynamical system to the state of criticality is termed order parameter. This parameter in the frame of natural time analysis of seismicity is just the quantity $\kappa_{1}$ as explained in detail in [33] as well as in pp. 249–254 of [4] and it is just the variance $\kappa_{1}\left(\equiv \langle \chi^{2}\rangle -{\langle \chi \rangle }^{2}\right)$ of natural time χ weighted for the normalized energy of each EQ, which results from the analysis of a seismic catalogue. It was shown [32] that this order parameter of seismicity exhibits a unique change approximately at the date at which SES activities have been reported to initiate. Specifically it was found [32] that the fluctuations of the order parameter $\kappa_{1}$ of seismicity in Japan exhibit a clearly detectable minimum approximately at the time of the initiation of the SES activity observed by Uyeda et al. [14] almost two months before the onset of the volcanic-seismic swarm activity in 2000 in the Izu Island region, Japan. Such minima of the order parameter of seismicity have also been found [34] a few months before the occurrence of all six shallow EQs with magnitudes $M_{JMA}\geq 7.6$ (where $M_{JMA}$ is the EQ magnitude reported by the Japan Meteorological Agency, JMA) that occurred in Japan during the 27 year period from 1 January 1984 until 11 March 2011 (which is the date of the occurrence of the M9 Tohoku EQ) as follows: The seismicity of Japan was analysed in natural time from 1 January 1984 until 11 March 2011 using sliding natural time windows comprising 200 or 300 consecutive EQs with $M_{JMA}\geq 3.5$, i.e., a number of EQs which is close to the average number of events that would occur in a few months (which is on average the lead time of SES activities). Fifteen distinct minima—deeper than a certain threshold—were identified a few months before large EQs, including all the six shallow EQs with $M_{JMA}\geq 7.6$ that occurred in Japan during this period. It was later shown [35] by means of Monte-Carlo calculation as well as by the receiver operating characteristics (ROC) technique that the probability to obtain the above result by chance is of the order of $10^{-5}$. In addition, Varotsos et al. [32] showed that the two phenomena (initiation of SES activity and minimum of the order parameter fluctuations) are also linked closely in space. This opened the window for a reliable estimation of the epicentral area of an impending EQ, as subsequently confirmed by the estimation [36] of the epicentral area for all shallow major mainshocks of magnitude 7.6 or larger that occurred in Japan during the aforementioned period 1984–2011.

**Figure 1:**
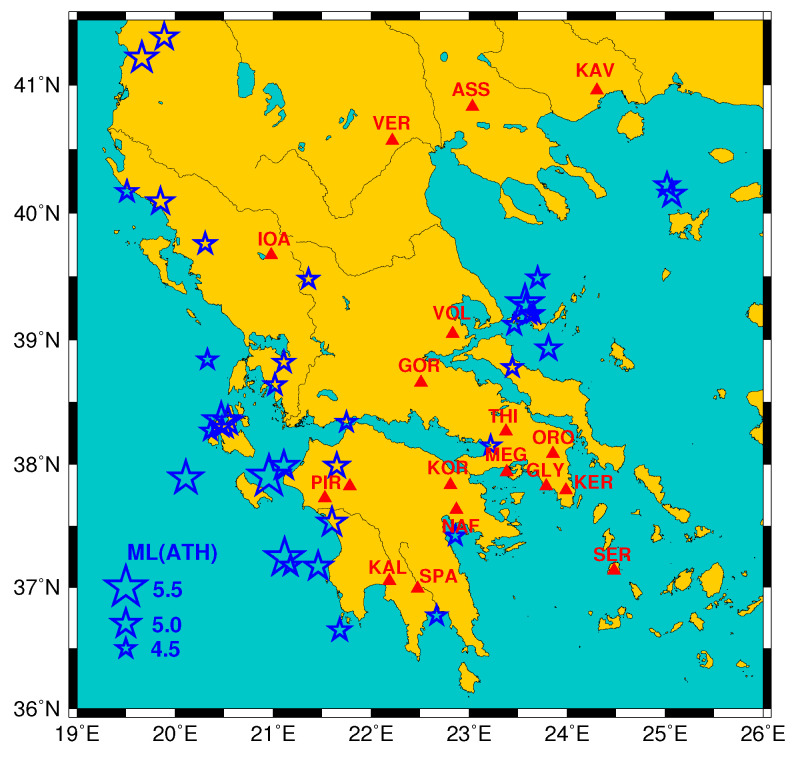
The measuring sites (IOA:Ioannina, PIR:Pirgos, KAL:Kalamata, VER:Veria, SPA:Sparta, GOR:Gorgopotamos, KOR:Korinthos, VOL:Volos, NAF:Nafplio, ASS:Assiros, THI:Thiva, MEG:Megara, GLY:Glyfada, ORO:Oropos, KER:Keratea, KAV: Kavala, SER:Serifos) of the telemetric network operating [3] in Greece during the period from 1 April 1987 to 30 November 1989 are depicted by the red triangles while the blue stars correspond to the epicentres of the earthquakes (EQs) with local earthquake magnitude reported by GI-NOA (ML(ATH)) ≥4.5. The dates of occurrence and their magnitudes are shown in Figure 2.

It is the scope of this paper to investigate the statistical significance of the aforementioned precursory phenomena and in particular the anomalous variations observed in the Earth’s electric and magnetic field before EQs. Along these lines we will study two independent datasets, one from Greece and another one from Japan. Specifically, the dataset from Greece comprises all the SES reported during the almost 2$\frac{1}{2}$ year period from 1 April 1987 until 30 November 1989 that possibly preceded EQs within the area $N_{36.0}^{41.5}E_{19.0}^{26.0}$ (see Figure 1). The selection of this early dataset is made for two reasons: First, the statistical significance of the data set has been previously studied in other works (e.g., [37]) showing that SES predictions are far beyond chance but with conventional techniques. Second, during this early period, predictions based on SES were issued when the expected EQ magnitude was 5 or larger, while later, i.e., after mid-1990s the European Advisory Committee for EQ prediction of the Council of Europe recommended (see p. 102 of [38]) that predictions should be issued *only* for large magnitudes, i.e., with local magnitudes $M_{L}\geq 5.5$ and hence Ms(ATH) ≥6.0, thus the number of predictions based on SES became considerably smaller.

As for the dataset from Japan, it comprises the geomagnetic data and local EQs registered in Kakioka (KAK) station in Japan during the 10 year period 2001–2010, which has been recently studied by Han et al. [29]. They utilized Molchan’s error diagram [39] to evaluate whether geomagnetic changes contain precursory information beyond chance in a strikingly similar fashion with the procedure followed in [40,41] which also employed Molchan’s diagram to show that the predictions issued on the basis of SES during the period 1987–1995 were appreciably better than random score (see Figure 2 of [40]). Han et al. [29] concluded that their magnetic field precursory variations perform better than chance when the lead time Δ is around one week and the alarm window *L* is less than four d *or*Δ is 13–14 d and *L* is less than one week. Interestingly, the former value almost coincides with the lead time reported for the single SES in Greece and the quantity Δ+L of the latter agrees only with the minimum (≈22 d) of the lead time of SES activities observed both in Greece and Japan (cf. in these cases Δt may be appreciably longer, i.e., around a few months or so). We report for example two such cases from Japan (cf. several similar cases in Greece are reported in [5]): First, the magnetic (and electric) field change before the volcanic-seismic swarm activity in 2000 in the Izu Island region started more than two months before [14,15]. (This lead time was attributed by Han et al. [29] to the volcanic-seismic nature in the Izu Island activity, but they did not consider that similarly long lead times had been recorded repeatedly in Greece for tectonic EQs since the early 1990s [5,42].) The same holds for the magnetic field anomaly observed before the M9 Tohoku EQ since it appeared (e.g., [43,44]) during the week around 5 January 2011 and the EQ occurred in 11 March 2011 (cf. on 5 January 2011 natural time analysis has revealed [34] that the minimum of the order parameter of seismicity has been observed which, as mentioned, may allow an estimation of both the time of occurrence [45] and the epicentral location [46] of the forthcoming mainshock). Thus, in short, Han et al. [29] claim that the maximum lead time is Δ+L=22 d which however does not agree either with longer lead times reported in Greece or with the aforementioned established examples in Japan for the cases of Izu and Tohoku. The present work sheds light on this disagreement.

In the present study, we solely employ modern statistical tools like the event coincidence analysis (ECA) [47] and the receiver operating characteristics (ROC) technique [48]. We clarify in advance that here we restrict ourselves to the probabilities to obtain by chance the results referring solely to the occurrence times of the events while the relevant calculation for achieving all the parameters of an impending EQ (time, epicenter, and magnitude) should also consider the probabilities to obtain the epicentral area and the magnitude of the impending EQs, e.g., [49,50].

## 2. Modern Statistical Tools. Background

### 2.1. Event Coincidence Analysis (ECA)

ECA has been designed [47] to quantify the *strength*, *directionality* and *time lag* of the statistical relations between event series. According to Donges et al. [47]: the method was introduced in a less general setting to study possible statistical interrelationships between nonlinear regime shifts in African peleoclimate during the past 5 million years and events in hominin evolution such as the appearance and disappearance of species [51]. ECA considers [47] a pair of two event time series *A* and *B* defined as two ordered event sets with timings $\left\{t_{1}^{A},t_{2}^{A},\ldots ,t_{N_{A}}^{A}\right\}$ and $\left\{t_{1}^{B},t_{2}^{B},\ldots ,t_{N_{B}}^{B}\right\}$, respectively. Hence, we have $N_{A}$ events of type A and $N_{B}$ events of type B. Both event series are assumed to cover a time interval $\left[t_{0},t_{f}\right]$, i.e., $t_{0}\leq t_{1}^{A},t_{1}^{B}$ and $t_{f}\geq t_{N_{A}}^{A},t_{N_{B}}^{B}$, and ECA is based on counting (possibly lagged) coincidences between events of different types. In this context [52], B type events are considered as possibly influencing the timings of A type events, and not vice versa. The assumption to be quantified and tested by ECA is that the events in B precede the events in A. This is made by introducing an instantaneous coincidence if two events with timings $t_{i}^{A}$ and $t_{j}^{B}$ ($t_{i}^{A}\geq t_{j}^{B}$) are closer in time than a coincidence interval ΔT, that means

(1) $$t_{i}^{A}-t_{j}^{B}\leq \Delta T,$$

and generalizing it to a lagged coincidence if

(2) $$\left(t_{i}^{A}-\tau \right)-t_{j}^{B}\leq \Delta T,$$

holds, where τ≥0 is the *time lag* parameter. In order to quantify the *strength* of the interrelations between the two event series, two variants of the coincidence rate addressing B type events as either precursors or trigger have been introduced [47]: The precursor coincidence rate

(3) $$r_{p}\left(\Delta T,\tau \right)=\frac{1}{N_{A}}\sum_{i=1}^{N{}_{A}}\Theta \left[\sum_{j=1}^{N_{B}}1_{\left[0,\Delta T\right]}\left(t_{i}^{A}-\tau -t_{j}^{B}\right)\right],$$

where Θ(x) is the Heaviside unit step function (equal to 0 for x≤0 and 1 for x>0) and $1_{I}\left(x\right)$ is the indicator function for the interval *I* (equal to 1 for x∈I and 0 otherwise), measures the fraction of A type events that are preceded *at least* by one B type event (i.e., multiple B type events within ΔT are counted once) and the trigger coincidence rate

(4) $$r_{t}\left(\Delta T,\tau \right)=\frac{1}{N_{B}}\sum_{j=1}^{N{}_{B}}\Theta \left[\sum_{i=1}^{N_{A}}1_{\left[0,\Delta T\right]}\left(t_{i}^{A}-\tau -t_{j}^{B}\right)\right],$$

measures the fraction of B type events that are followed by at least one A type event (i.e., multiple A type events within ΔT are counted once). The distinction between precursor and trigger coincidence rates allows the introduction of a notion of *directionality* while the parameter τ explicitly takes into account lagged interrelations between B type and A type events. Based on these two coincidence rates and appropriate assumptions (for example for the inter-event time distributions [47]) various statistical tests can be made to examine whether B type events are precursors to A type events for a risk enhancement test [53] or whether B type events are triggers for A type events (trigger test [53]). Typical examples are the climate-related disasters as risk enhancement factor for armed-conflicts in ethnically fractionalized countries [53] or the role of flood events as triggers of epidemic outbreaks [47]. Here, we employed the function CC.eca.es of the CoinCalc package [52] for R [54] that implements ECA and selected the (default) Poisson test. The reason behind this selection, although it is known that EQs appear in sequences due to aftershocks, was that the magnitude range of the EQs (that define A type events) in most of the studies here barely exceeds one magnitude unit while according to Båth law  [4,55,56,57,58] the difference in magnitude between the mainshock and its largest aftershock is approximately 1.2 magnitude units. In other words, in the data presented in Section 3 below, it is improbable that aftershocks are used for the determination of A type events (cf. only in Section 3.2 and when we consider all the 50 EQs of Table 2 of [59] there is a possibility that a single aftershock, i.e., a M4.8 EQ on 16 November 2005, is included; if so the mainshock, i.e., the M6.3 EQ on 19 October 2005, had occurred 28 days before. Such an inter-event time, however, is not rare in Table 2 of [59]).

### 2.2. ROC Analysis

ROC analysis is a modern technique [48] that depicts the trade off between hit rates and false alarm rates in a conceptually simple way. It has been recently applied [35,60,61,62,63,64,65,66,67] for estimating the predictability of various complex systems and as such it might be useful and complementary to ECA, which was mentioned in the previous subsection.

Suppose that we have in total *N* events out of which *P* are strong and important to be predicted and Q=N−P of which are not as strong (for example in the case of EQs one may consider as strong an EQ of magnitude *M* exceeding some threshold $M_{thres}$, i.e., $M\geq M_{thres}$, which might be also called “strong event”, while smaller EQs may be considered as “non-events”). Suppose also that before the occurrence of each event either strong or not, a quantity labeled ϵ is estimated based on some prediction algorithm. If $\epsilon \geq \epsilon_{t}$, where $\epsilon_{t}$ is some threshold value, we assume that the forthcoming event will be of the *P* class and if $\epsilon <\epsilon_{t}$ a *Q* class event is expected. Upon the occurrence of the forthcoming event, four situations may appear: (a) If $\epsilon \geq \epsilon_{t}$ and the event is *P* class we have a successful prediction called True Positive (TP); (b) if $\epsilon <\epsilon_{t}$ and the event is *Q* class we have a successful prediction called True Negative (TN); (c) if $\epsilon \geq \epsilon_{t}$ and the event is *Q* class we have a False Positive (FP) prediction; and (d) if $\epsilon <\epsilon_{t}$ and the event is *P* class we have a False Negative (FN) prediction.

ROC analysis is based on a graph in which we plot the True Positive rate (TPr), or hit rate (*H*), which is the number |TP| of TPs divided by *P*:

(5) $$H\equiv \frac{\left|TP\right|}{P}=\frac{\left|TP\right|}{\left|TP|+|FN\right|},$$

versus the False Positive rate (FPr), or false alarm rate (*F*), which is the number |FP| of FPs divided by *Q*:

(6) $$F\equiv \frac{\left|FP\right|}{Q}=\frac{\left|FP\right|}{\left|FP|+|TN\right|}.$$

A ROC curve is obtained as we vary $\epsilon_{t}$ and plot TPr as a function of FPr. Thus, each prediction scheme (e.g., selection of $\epsilon_{t}$) may be considered as a point in the ROC curve. If the predictor ϵ is decided at random, the ROC curve will scatter close to the diagonal (which will result if both *P* and *Q* tend to infinity) but for real problems it fluctuates as *P* and *Q* vary. The statistical significance of a ROC curve depends on the area above the horizontal axis (TPr = 0) and under the curve [68]. By assuming an appropriate set of ellipses, called *k*-ellipses, we may estimate [66] the *p*-value to obtain by chance a point on the ROC. Here, we employed the computer code VISROC [66] that allows the visualization of this *p*-value to obtain by chance a point on the ROC graph and hence this enables us to perform statistical tests on whether a prediction scheme outperforms chance.

## 3. Results

### 3.1. Greece

**Figure 2:**
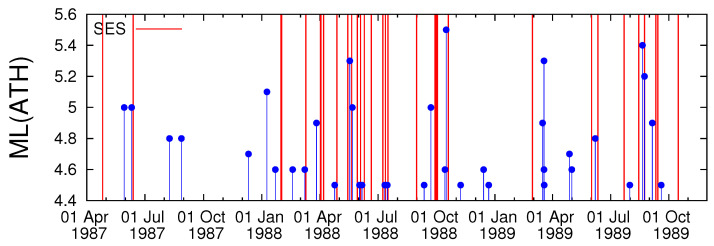
The EQ local magnitude ML(ATH) (blue candlesticks ending at circles) reported by GI-NOA, available from http://www.gein.noa.gr/services/fullcatalogue.php, for all the seismic events that occurred during the period 1 April 1987 to 30 November 1989 within the area $N_{36.0}^{41.5}E_{19.0}^{26.0}$ with ML(ATH) ≥4.5. The red vertical lines correspond to the dates that SES were recorded (Table 1, see also Appendix I of [69]) and led to predictions of magnitude class 5 EQs according to Tables 1, 2 and 4 of [5].

**Figure 3:**
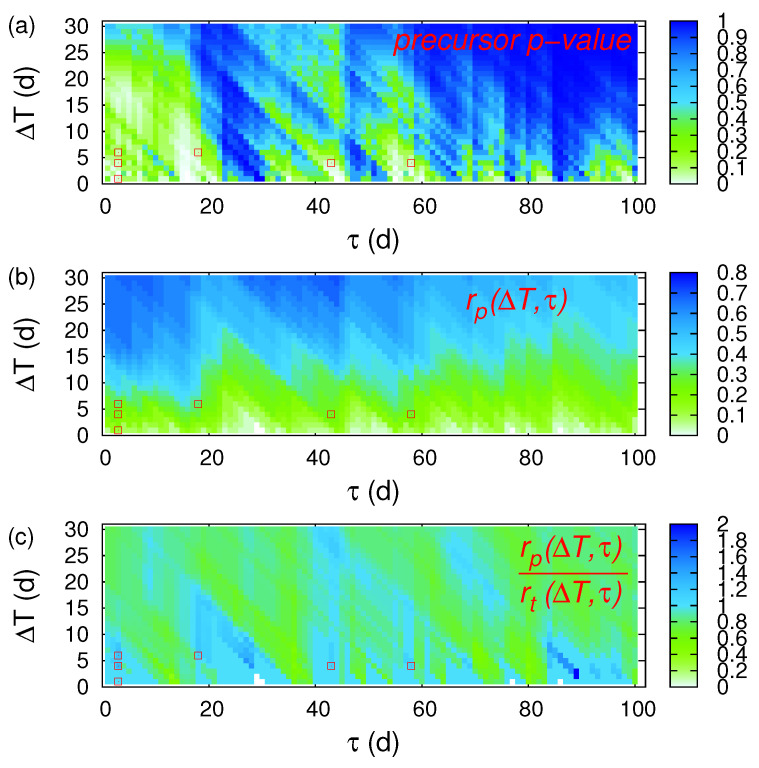
Event Coincidence Analysis (ECA) from 1 April 1987 to 30 November 1989 in Greece for the Seismic Electric Signals (SES) discussed in Section 3.1. The colours indicate (**a**) the precursor *p*-value; (**b**) the precursor coincidence rate; and (**c**) the ratio of the precursor over the trigger coincidence rate. These values were obtained by applying CoinCalc [52] as discussed in Section 2.1. The red squares indicate the statistically significant cases of (ΔT,τ) that apart from having a precursory *p*-value smaller than 5%, they also exhibit a precursory coincidence rate exceeding the trigger coincidence rate.

In Table 1 we give all the SES reported [3,5,69] during the period from 1 April 1987 until 30 November 1989. The dates of all these SES are plotted in Figure 2 as vertical red lines along with the local magnitude ML(ATH) (≥4.5) of the EQs that occurred within the area $N_{36.0}^{41.5}E_{19.0}^{26.0}$ reported by GI-NOA (for their epicentres see Figure 1). By applying ECA between the event series corresponding to the occurrence dates of these EQs (event time series *A*, see Section 2.1) and the event series of the SES dates of Table 1 (event time series *B*, see Section 2.1), we find (see Figure 3) that for τ=3 d and ΔT=4 d the precursor *p*-value to obtain by chance the real interconnection between the event time series *A* and *B* is 2.7% (while the corresponding precursor coincidence rate, which equals the hit rate, is 26.5%). In other words, the SES are statistically significant precursors to Ms(ATH) ≥5.0 EQs. If we increase ΔT to 6 d, we obtain a *p*-value 4.5% with $r_{p}(\Delta T=6$ d, τ=3 d) = 32.4% also pointing to statistical significance. Upon focusing on the cases when $r_{p}$ exceeds $r_{t}$ (see red squares in Figure 3c), we also found that SES are statistically significant precursors to the EQs depicted in Figure 2 in the following three cases: First, the *p*-value results in 4.9% when using τ=18 d and ΔT=6 d with $r_{p}(\Delta T=6$ d, τ=18 d) = 32.3%; second, the *p*-value results in 3.4% when using τ=43 d and ΔT=4 d with $r_{p}(\Delta T=4$ d, τ=43 d) = 26.5%; third, the *p*-value results in 3.7% when using τ=58 d and ΔT=4 d with $r_{p}(\Delta T=4$ d, τ=58 d) = 26.5%. Additional SES activities verifying these three cases can be found in [5,42,70,71]. By summarizing, we found that SES are statistical significant precursors to EQs for the following four distinct time periods [τ,τ+ΔT]: 3 to 9 d, 18 to 24 d, 43 to 47 d and 58 to 62 d which are strikingly reminiscent of the finding [3,4,5,42] that single SES have a Δt≤ 11 d while for SES activities the minimum lead time Δt is around a few weeks and the maximum $5\frac{1}{2}$ months, as mentioned in the introduction. Since in a finite sample, as studied here, there is always a chance of false positive significance in a certain fraction of individual tests, we also repeated ECA 30 times with the SES dates randomly chosen. Out of these 30 cases studied, only three of them led to smaller than six and larger than zero statistically significant pairs (ΔT,τ) for which $r_{p}$ exceeds $r_{t}$. Moreover, in all these three cases the obtained τ values included consecutive τ’s (e.g., in one such case the τ-values 10, 11, and 49 were obtained). In the real case studied above, however, there were no consecutive τ values which points to the existence of a physical mechanism rather than a chancy correlation. In other words, the probability of obtaining by chance the four distinct periods [τ,τ+ΔT]: 3 to 9 d, 18 to 24 d, 43 to 47 d and 58 to 62 d is below 1/30 ≈3.3%.

**Figure 4:**
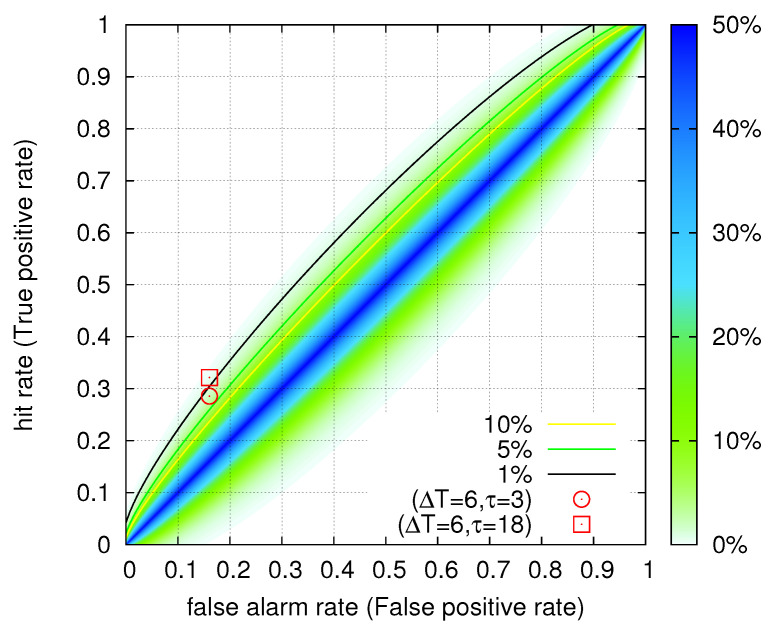
Receiver Operating Characteristics (ROC) analysis for SES from 1 April 1987 to 30 November 1989 in Greece by considering consecutive one week periods. The colour contours indicate [66] the probability to obtain by chance a point in the ROC plane when 28(=*P*) events out of 140(=P+Q) are to be predicted (see Section 2.2). The operation points (ΔT=6 d, τ=3 d) (circle) and (ΔT=6 d, τ=18 d) (square) discussed in Section 3.1 have *p*-values 2.0% and 0.5%, respectively.

Based on these results, we also applied the ROC technique by dividing the study period into consecutive weeks (i.e., a time interval corresponding to the larger two ΔT values of the previous paragraph). Based on whether a certain week included a ML(ATH) ≥ 4.5 EQ (as depicted in Figure 2) or not, we decided if this week had to be predicted (strong event, see Section 2.2) or not. This resulted in 28 weeks including such EQs out of the 140 weeks in total. The SES dates were also segmented (coarse grained) into weeks for which an alarm was issued or not. This procedure has led to 27 alarm weeks. The operation points for example of the two cases: (ΔT=6 d, τ=3 d) and (ΔT=6 d, τ=18 d) out of the four cases identified by ECA and discussed in the previous paragraph are shown by the circle and the square, respectively, in the ROC depicted in Figure 4. The *p*-values to obtain these results by chance are 2.0% and 0.5%, respectively, which are statistically significant.

### 3.2. Japan

**Figure 5:**
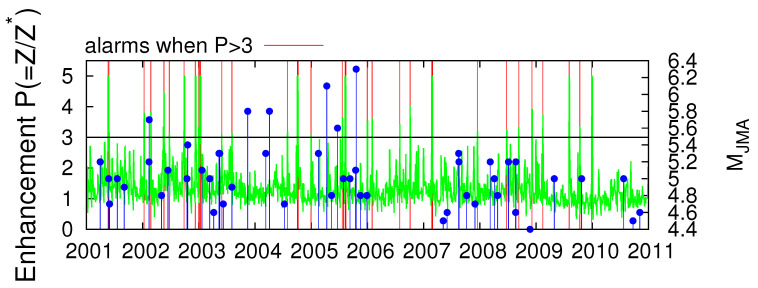
The energy enhancement parameter P (green) as reported in Figure 2a of Han et al. [29] together the EQ magnitude $M_{JMA}$ (blue candlesticks ending at circles, right scale) reported by Japan Meteorological Agency (JMA) for the 50 EQs of Table 2 of [59] that occurred within 100 km from Kakioka (KAK). The red vertical lines correspond to the dates (see Table 2) when P exceeds the threshold value $P^{*}=3$, i.e., when P >3.

Peng et al. [59] defined the energy enhancement parameter P, as the ratio of the observed energy (Z) at Kakioka (KAK) station in the vertical component of the magnetic field in the ULF region, i.e., at around [28] 0.01 Hz, over its modelled value ($Z^{*}$) estimated on the basis of the measurements made at a remote (≈1000 km away) station [59]. This parameter, given in Figure 2a of Han et al. [29], is plotted for the 10-year period 2001–2010 in Figure 5 together with the EQ magnitude $M_{JMA}$ for 50 sizeable EQs (see Table 2 of [59]) that occurred within 100 km from KAK, which according to [29,59] could have been preceded by energy enhancements in the ULF variation of the vertical component of the geomagnetic field. Here, following [29] we will assume that when P exceeds a threshold value $P^{*}$ an alarm for an impending EQ is issued. For example, in Figure 5 the red vertical lines correspond to the dates for which P exceeds the threshold value $P^{*}=3$, i.e., when P >3. These dates as a first approximation have been selected as a coarse-grained average over three-day periods from Figure 2a of Han et al. [29] and lead to the 34 alarm dates given in Table 2.

**Figure 6:**
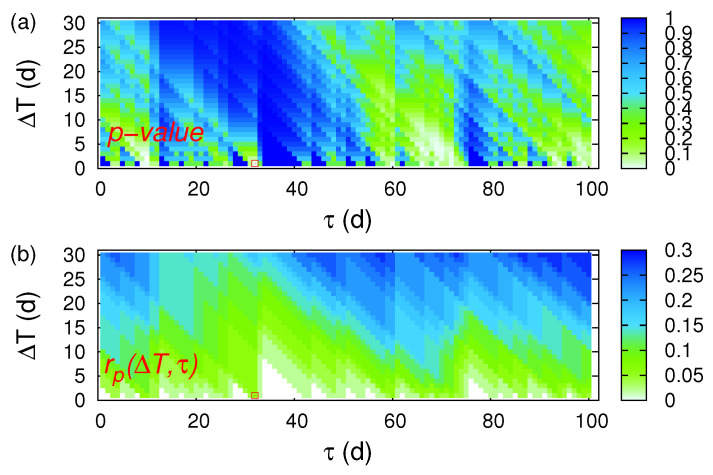
ECA for the 10-year period 2001–2010 in Japan when studying the magnetic field data shown in Figure 5. The colours indicate (**a**) the precursor *p*-value and (**b**) the precursor coincidence rate which were obtained by applying CoinCalc [52]. The red square indicates the single statistically significant combination (ΔT=1 d, τ=32 d) that apart from having a precursory *p*-value smaller than 5%, it also exhibits a precursory coincidence rate exceeding the trigger coincidence rate.

We start with ECA for the 10-year period 2001–2010 (3652 d) and consider the event series of the 50 EQs shown in Figure 5 as event time series *A* and the event series comprising the 34 alarm dates shown in Table 2 as event series *B*. We investigated again the cases for which the precursor coincidence rate exceeds the trigger coincidence rate (see the red square in Figure 6), since we want to perform a risk enhancement test. Such an analysis resulted in one statistically significant solution (ΔT=1 d, τ=32 d) with *p*-value 1.2%. The corresponding hit rate is $r_{p}(\Delta T=1$ d, τ=32 d) = 6%.

**Figure 7:**
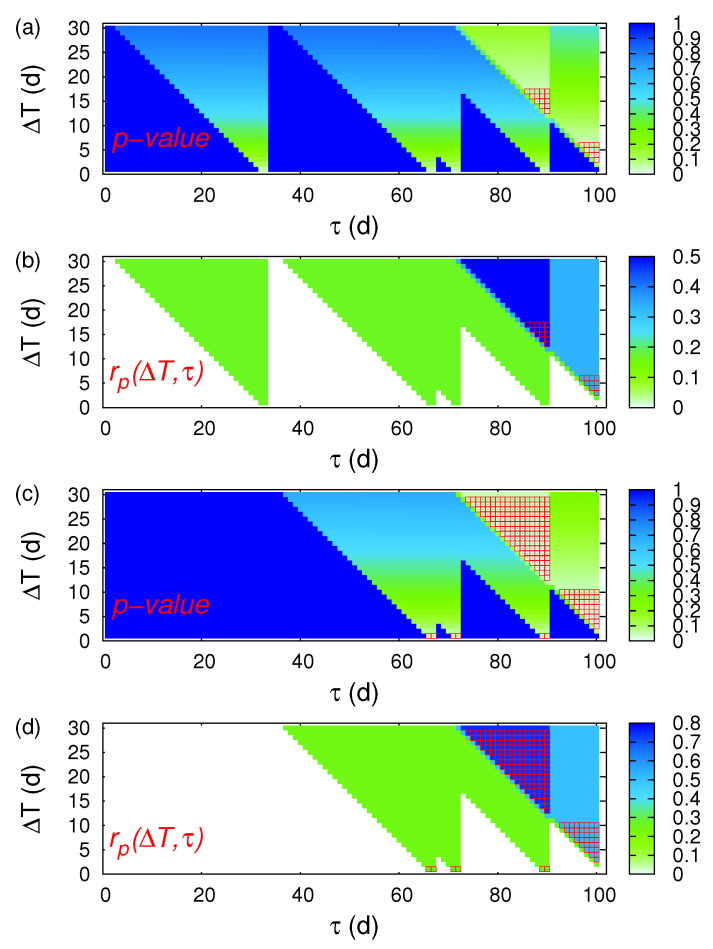
ECA for the 10-year period 2001–2010 in Japan when studying the magnetic field data shown in Figure 5 and focusing on the prediction of large magnitude EQs. The colours indicate in (**a**,**c**) the precursor *p*-value and in (**b**,**d**) the precursor coincidence rate which were obtained by applying CoinCalc [52]. Panels (**a**,**b**) correspond to $M_{JMA}\geq 5.5$ while (**c**,**d**) to $M_{JMA}\geq 5.8$. The red squares indicate the statistically significant cases of (ΔT,τ) that apart from having a precursory *p*-value smaller than 5%, they also exhibit a precursory coincidence rate exceeding the trigger coincidence rate.

Hereafter, we focus on the large magnitude EQs (see Figure 7) searching for statistically significant cases with the highest hit rate and the smallest *p*-value. We first investigate the EQs with $M_{JMA}\geq 5.5$ (six EQs in total). Such a statistically significant solution was obtained for (ΔT=13 d, τ=90 d) with a *p*-value 2.4% and $r_{p}(\Delta T=13$ d, τ=90 d) = 50%. Secondly, we investigate the EQs with $M_{JMA}\geq 5.8$ (four EQs in total). Such a statistically significant solution was again obtained for (ΔT=13 d, τ=90 d) with a *p*-value 0.6% and $r_{p}(\Delta T=13$ d, τ=90 d) = 75%.

**Figure 8:**
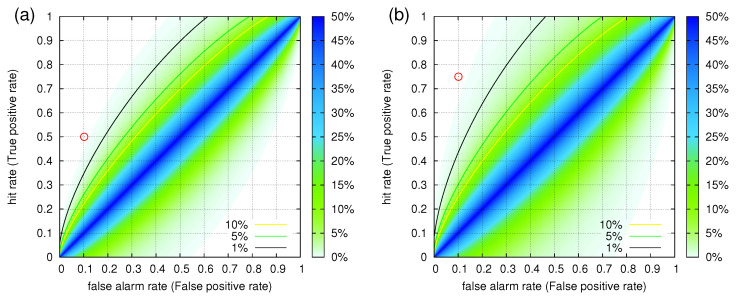
ROC analysis when studying the magnetic field data during the 10-year period 2001–2010 in Japan by considering consecutive periods of two weeks. The colour contours indicate [66] the probability to obtain by chance a point in the ROC plane. The operation points for (ΔT=13 d, τ=90 d) are indicated by circles when they focus on the prediction of the $M_{JMA}\geq 5.5$ (**a**) and $M_{JMA}\geq 5.8$ (**b**) EQs of Table 2 of Peng et al. [59] discussed in Section 3.2. In both cases, the estimated [66] *p*-values are 0.1%.

We now turn to ROC analysis. Since the results of the previous paragraph point to the importance of ΔT=13 d and hence a two-week period, we divided the study period of 3652 d into 261 consecutive 14 d periods. Based on whether a certain two-week period included a $M_{JMA}\geq$ 5.5 EQ or not, we decided if this period had to be predicted (strong event, see Section 2.2) or not. This resulted in six periods including such EQs out of the 261 in total. The alarm dates were also segmented (coarse grained) into two-week periods for which an alarm was issued or not. This procedure led to 29 alarms, which when considering that three alarms were followed by at least one EQ, we obtain an FPr F=10.2% while the hit rate is H=50% as mentioned. The operation point of the pair (ΔT=13 d, τ=90 d) discussed in the previous paragraph is shown by the circle in the ROC depicted in Figure 8a. The *p*-value to obtain this result by chance is 0.1%. By applying the same procedure to predict $M_{JMA}\geq$ 5.8 EQs, we obtain the operation point indicated by the circle in the ROC analysis shown in Figure 8b. The *p*-value to obtain this result by chance is again found to be 0.1%.

## 4. Discussion

We first comment on the results obtained in Section 3.1 in which by means of ECA, we found that SES recorded since 1980s in Greece are statistically significant precursors to EQs for four distinct time periods 3 to 9 d, 18 to 24 d, 43 to 47 d and 58 to 62 d. The first time period agrees with the experimental results deduced for single SES (Δt≤11 d) in Greece, and the other three time periods correspond to the lead times identified for SES activities in Greece. Examples are: For the 18 to 24 d period, the M6.6 Grevena-Kozani EQ on 13 May 1995 that was preceded [4,24,25,38] by two SES activities on 18 and 19 April 1995; for the period 43 to 47 d, the destructive M6.5 Egion EQ on 15 June 1995 that was preceded [4,24,38] by an SES activity on 30 April 1995; for the period 58 to 62 d, the M6.6 Leonidion EQ on 6 January 2008 that was preceded [4,70] by an SES activity on 7 November 2007. We recall that during the almost 40-year period from the 1980s to 2018 in Greece, several large magnitude EQs 5.5<M<7 occurred and their preceding SES activities had lead times up to $5\frac{1}{2}$ months [3,4,5,42,71]. As mentioned in the introduction, since the mid-1990s SES predictions are issued when the expected magnitude is Ms(ATH) ≥6.0. A study concerning such predictions for the period 2001 to 2011 can be found in Table 7.1 of [4]. The results that are summarized in Section 7.3 of [4] show that 15 out of the 16 EQs with Ms(ATH) ≥6.0 where preceded by SES. This points to the direction that if we consider higher magnitude thresholds, better results are expected. In the same context, it would be interesting to study the existing geoelectric records and vary the threshold for the identification of SES activities so that the operation points in the ROC diagram of Figure 4 can be extended to ROC curves. This way an optimal operation can be selected. Such a study, however, falls beyond the scope of the present paper.

As for the results of ECA in Japan, it is striking that they do not reveal any of the two time periods, i.e., (Δ=1 week, L<4 d) and (Δ= 13–14 d, L<1 week), deduced by Han et al. [29] from the analysis of the Japanese magnetic field data during 2001–2010. To the contrary, the application of ECA to Japanese data resulted in a lead time of around one month, i.e., 32 to 33 d, and in particular for the six large magnitude EQs with $M_{JMA}>5.5$ during 2001–2010 it led to even longer lead times around $3\frac{1}{2}$ months. Quite interestingly, these lead times identified by ECA agree with experimental results obtained in Greece, but have not been identified by Han et al. [29] (for example the M6.5 Andravida EQ in Greece on 8 June 2008, was preceded [4,70] by an SES activity that initiated on 29 February 2008). These new findings could be attributed to the superiority of the modern statistical tools employed in the present study.

We now comment on the point made by Han et al. [29] that a lead time of around one week can be established as an optimal strategy for earthquake prediction. As we have shown in Section 3.2, such a lead time did not result from the 10-year (2001–2010) magnetic field data analysed by Han et al. [29]. A procedure to achieve a time window of around one week for the occurrence of an impending EQ is to apply the following procedure based on natural time analysis: Just after the initiation of the SES activity, we analyse in natural time the EQs that occur in the candidate epicentral area (estimated either on the basis of the SES properties [70] or with the procedure developed in [36,46]) and compute the $\kappa_{1}$ value upon the occurrence of each EQ. Once this value approaches 0.070, e.g., see [4,9,70,72], the mainshock occurs within one week or so. Such an application for the M9 Tohoku EQ that occurred in 11 March 2011 in Japan has already appeared in [46]. Concerning this mega-earthquake, an interesting application of natural time analysis to ULF magnetic field variations can be found in [73].

Finally, we note that the two example cases presented here correspond to the most seismic active regions of the world (Japan) and of Europe (Greece). As such, they constitute representative examples which may find useful application to other seismic prone regions. Of course, the establishment and operation of geoelectric and/or geomagnetic measurement networks together with similar statistical studies will provide the ultimate test.

## 5. Summary and Conclusions

Here, we employed two modern statistical tools, i.e., ECA and ROC, to revisit the statistical significance of the anomalous changes of the Earth’s electric and magnetic field as precursory to EQs. Two independent datasets (first, electric field data from Greece since the 1980s and second, magnetic field data from Japan during 2001–2010 which is alternatively termed ULF seismo-magnetic phenomena) were studied and we found that they exhibit precursory information far beyond chance. In addition, our results show that in both datasets the corresponding lead times are comparable to those determined experimentally in Greece since the 1980s. Quite interestingly, upon restricting ourselves to magnetic field changes before major EQs in Japan, we find that ECA succeeds in identifying their correct lead times, which are around $3\frac{1}{2}$ months, in agreement with comparable experimental values reported in Greece. On the other hand, previous conventional calculations for the analysis of Japanese data by other studies, e.g., [29], obtained appreciably shorter lead times (around one week and two weeks).

## Figures and Tables

**Table 1:** SES recorded during the period from 1 April 1987 until 30 November 1989 (see Tables 1, 2 and 4 of [5], see also [69]). The sites of the measuring stations are shown in Figure 1.

Measuring Station	Date (YYYYMMDD)
PIR	19870426
PIR	19870613
ASS, KAV, KER	19880131
KAV, KER	19880201
IOA	19880310
IOA	19880402
KER	19880403
IOA	19880407
IOA	19880428
IOA	19880515
IOA	19880521
IOA	19880530
IOA	19880604
KER	19880610
IOA	19880621
PIR	19880709
KOR	19880713
NAF	19880717
IOA	19880831
IOA	19880929
IOA	19880930
IOA	19881001
IOA	19881002
IOA	19881003
IOA	19881020
IOA	19890301
IOA	19890602
IOA	19890612
KER	19890723
IOA	19890815
IOA	19890824
IOA	19890911
ASS	19890914
IOA	19891016

**Table 2:** The dates for which the energy enhancement of the vertical component of the geomagnetic field P is greater than or equal to 3.0, see Section 3.2.

No	Date (YYYYMMDD)
1	20010521
2	20010527
3	20020110
4	20020223
5	20020519
6	20020623
7	20020927
8	20021209
9	20021231
10	20030105
11	20030111
12	20030530
13	20030804
14	20040729
15	20041002
16	20041007
17	20041230
18	20050721
19	20050808
20	20050813
21	20051230
22	20060131
23	20060730
24	20061005
25	20070222
26	20070227
27	20071215
28	20080623
29	20080910
30	20081204
31	20090213
32	20090803
33	20091011
34	20091228

## Abbreviations

The following abbreviations are used in this manuscript:

ATH	Athens
d	days
ECA	Event coincidence analysis
EQ	Earthquake
FN	False negative
FP	False positive
FPr	False positive rate
GI-NOA	Institute of Geodynamics of the National Observatory of Athens, Greece
JMA	Japan Meteorological Agency
KAK	Kakioka, Japan, geomagnetic station (see [59])
ML(ATH)	Local earthquake magnitude reported by GI-NOA
SES	Seismic electric signals
TN	True negative
TP	True positive
TPr	True positive rate
ROC	Receiver operating characteristics
ULF	Ultra-low frequency

## References

[1] Varotsos P., Alexopoulos K. (1984). Physical Properties of the variations of the electric field of the earth preceding earthquakes, I. Tectonophysics.

[2] Varotsos P., Alexopoulos K. (1984). Physical Properties of the variations of the electric field of the earth preceding earthquakes, II. Tectonophysics.

[3] Varotsos P., Lazaridou M. (1991). Latest aspects of earthquake prediction in Greece based on Seismic Electric Signals. Tectonophysics.

[4] Varotsos P.A., Sarlis N.V., Skordas E.S. (2011). Natural Time Analysis: The New View of Time. Precursory Seismic Electric Signals, Earthquakes and other Complex Time-Series.

[5] Varotsos P., Alexopoulos K., Lazaridou M. (1993). Latest aspects of earthquake prediction in Greece based on Seismic Electric Signals, II. Tectonophysics.

[6] Lazaridou M., Varotsos C., Alexopoulos K., Varotsos P. (1985). Point-defect parameters of LiF. J. Phys. C Solid State.

[7] Uyeda S., Nagao T., Kamogawa M. (2009). Short-term earthquake prediction: Current status of seismo-electromagnetics. Tectonophysics.

[8] Varotsos P.A., Sarlis N.V., Skordas E.S. (2002). Long-range correlations in the electric signals that precede rupture. Phys. Rev. E.

[9] Varotsos P.A., Sarlis N.V., Skordas E.S., Lazaridou M.S. (2008). Fluctuations, under time reversal, of the natural time and the entropy distinguish similar looking electric signals of different dynamics. J. Appl. Phys..

[10] Varotsos P., Sarlis N.V., Skordas E.S., Uyeda S., Kamogawa M. (2011). Natural time analysis of critical phenomena. Proc. Natl. Acad. Sci. USA.

[11] Sarlis N., Lazaridou M., Kapiris P., Varotsos P. (1999). Numerical Model of the Selectivity Effect and ΔV/L criterion. Geophys. Res. Lett..

[12] Varotsos P., Sarlis N., Lazaridou M., Kapiris P. (1998). Transmission of stress induced electric signals in dielectric media. J. Appl. Phys..

[13] Uyeda S., Nagao T., Orihara Y., Yamaguchi T., Takahashi I. (2000). Geoelectric potential changes: Possible precursors to earthquakes in Japan. Proc. Natl. Acad. Sci. USA.

[14] Uyeda S., Hayakawa M., Nagao T., Molchanov O., Hattori K., Orihara Y., Gotoh K., Akinaga Y., Tanaka H. (2002). Electric and magnetic phenomena observed before the volcano-seismic activity in 2000 in the Izu Island Region, Japan. Proc. Natl. Acad. Sci. USA.

[15] Uyeda S., Kamogawa M., Tanaka H. (2009). Analysis of electrical activity and seismicity in the natural time domain for the volcanic-seismic swarm activity in 2000 in the Izu Island region, Japan. J. Geophys. Res..

[16] Orihara Y., Kamogawa M., Nagao T., Uyeda S. (2009). Independent component analysis of geoelectric field data in the northern Nagano, Japan. Proc. Jpn. Acad. Ser. B Phys. Biol. Sci..

[17] Orihara Y., Kamogawa M., Nagao T., Uyeda S. (2012). Preseismic anomalous telluric current signals observed in Kozu-shima Island, Japan. Proc. Natl. Acad. Sci. USA.

[18] Zlotnicki J., Kossobokov V., Le Mouël J.L. (2001). Frequency spectral properties of an ULF electromagnetic signal around the 21 July 1995, M = 5.7, Yong Deng (China) earthquake. Tectonophysics.

[19] Fan Y.Y., Du X.B., Zlotnicki J., Tan D.C., An Z.H., Chen J.Y., Zheng G.L., Liu J., Xie T. (2010). The Electromagnetic Phenomena Before the Ms8.0 Wenchuan Earthquake. Chin. J. Geophys..

[20] Huang Q. (2011). Retrospective investigation of geophysical data possibly associated with the Ms8.0 Wenchuan earthquake in Sichuan, China. J. Asian Earth Sci..

[21] Ramírez-Rojas A., Flores-Márquez E.L., Guzmán-Vargas L., Gálvez-Coyt G., Telesca L., Angulo-Brown F. (2008). Statistical features of seismoelectric signals prior to M7.4 Guerrero-Oaxaca earthquake (México). Nat. Hazards Earth Syst. Sci..

[22] Ramírez-Rojas A., Telesca L., Angulo-Brown F. (2011). Entropy of geoelectrical time series in the natural time domain. Nat. Hazards Earth Syst. Sci..

[23] Sarlis N.V., Varotsos P.A., Skordas E.S., Uyeda S., Zlotnicki J., Nagao T., Rybin A., Lazaridou-Varotsos M.S., Papadopoulou K.A. (2018). Seismic electric signals in seismic prone areas. Earthquake Sci..

[24] Varotsos P., Lazaridou M., Eftaxias K., Antonopoulos G., Makris J., Kopanas J., Lighthill M.J. (1996). Short term earthquake prediction in Greece by Seismic Electric Signals. The Critical Review of VAN: Earthquake Prediction from Seismic Electric Signals.

[25] Varotsos P.V., Sarlis N.V., Skordas E.S. (2003). Electric Fields that “arrive” before the time derivative of the magnetic field prior to major earthquakes. Phys. Rev. Lett..

[26] Varotsos P., Sarlis N., Lazaridou M., Kapiris P. (1996). A plausible model for the explanation of the selectivity effect of seismic electric signals. Pract. Athens Acad..

[27] Sarlis N., Varotsos P. (2002). Magnetic field near the outcrop of an almost horizontal conductive sheet. J. Geodyn..

[28] Hattori K., Han P., Yoshino C., Febriani F., Yamaguchi H., Chen C.H. (2013). Investigation of ULF Seismo-Magnetic Phenomena in Kanto, Japan During 2000–2010: Case Studies and Statistical Studies. Surv. Geophys..

[29] Han P., Hattori K., Zhuang J., Chen C.H., Liu J.Y., Yoshida S. (2017). Evaluation of ULF seismo-magnetic phenomena in Kakioka, Japan by using Molchan’s error diagram. Geophys. J. Int..

[30] Hattori K., Serita A., Yoshino C., Hayakawa M., Isezaki N. (2006). Singular spectral analysis and principal component analysis for signal discrimination of ULF geomagnetic data associated with 2000 Izu Island Earthquake Swarm. Phys. Chem. Earth Parts A/B/C.

[31] Hayakawa M., Hattori K., Ohta K. (2007). Monitoring of ULF (Ultra-Low-Frequency) Geomagnetic Variations Associated with Earthquakes. Sensors.

[32] Varotsos P.A., Sarlis N.V., Skordas E.S., Lazaridou M.S. (2013). Seismic Electric Signals: An additional fact showing their physical interconnection with seismicity. Tectonophysics.

[33] Varotsos P.A., Sarlis N.V., Tanaka H.K., Skordas E.S. (2005). Similarity of fluctuations in correlated systems: The case of seismicity. Phys. Rev. E.

[34] Sarlis N.V., Skordas E.S., Varotsos P.A., Nagao T., Kamogawa M., Tanaka H., Uyeda S. (2013). Minimum of the order parameter fluctuations of seismicity before major earthquakes in Japan. Proc. Natl. Acad. Sci. USA.

[35] Sarlis N.V., Skordas E.S., Christopoulos S.R.G., Varotsos P.A. (2016). Statistical Significance of Minimum of the Order Parameter Fluctuations of Seismicity Before Major Earthquakes in Japan. Pure Appl. Geophys..

[36] Sarlis N.V., Skordas E.S., Varotsos P.A., Nagao T., Kamogawa M., Uyeda S. (2015). Spatiotemporal variations of seismicity before major earthquakes in the Japanese area and their relation with the epicentral locations. Proc. Natl. Acad. Sci. USA.

[37] Hamada K. (1993). Statistical evaluation of the SES predictions issued in Greece: alarm and success rates. Tectonophysics.

[38] Varotsos P. (2005). The Physics of Seismic Electric Signals.

[39] Molchan G.M. (1991). Structure of optimal strategies in earthquake prediction. Tectonophysics.

[40] Varotsos P., Eftaxias K., Skordas E., Hadjicontis V., Lazaridou M. (1996). Reply to “Rebuttal to Replies I and II by Varotsos et al.” by F. Mulargia, W. Marzocchi and P. Gasperini. Geophys. Res. Lett..

[41] Varotsos P., Hadjicontis V., Eftaxias K., Skordas E., Lazaridou M. (1996). Reply to “Re-Rebuttal to the Reply of Varotsos et al.”, by F. Mulargia, W. Marzocchi, and P. Gasperini. Geophys. Res. Lett..

[42] Varotsos P., Alexopoulos K., Lazaridou-Varotsou M., Nagao T. (1993). Earthquake predictions issued in Greece by seismic electric signals since February 6, 1990. Tectonophysics.

[43] Xu G., Han P., Huang Q., Hattori K., Febriani F., Yamaguchi H. (2013). Anomalous behaviors of geomagnetic diurnal variations prior to the 2011 off the Pacific coast of Tohoku earthquake (Mw9.0). J. Asian Earth Sci..

[44] Han P., Hattori K., Xu G., Ashida R., Chen C.H., Febriani F., Yamaguchi H. (2015). Further investigations of geomagnetic diurnal variations associated with the 2011 off the Pacific coast of Tohoku earthquake (Mw 9.0). J. Asian Earth Sci..

[45] Skordas E., Sarlis N. (2014). On the anomalous changes of seismicity and geomagnetic field prior to the 2011 9.0 Tohoku earthquake. J. Asian Earth Sci..

[46] Varotsos P.A., Sarlis N.V., Skordas E.S. (2017). Identifying the occurrence time of an impending major earthquake: A review. Earthquake Sci..

[47] Donges J., Schleussner C.F., Siegmund J., Donner R. (2016). Event coincidence analysis for quantifying statistical interrelationships between event time series. Eur. Phys. J. Spec. Top..

[48] Fawcett T. (2006). An introduction to ROC analysis. Pattern Recogn. Lett..

[49] Varotsos P., Eftaxias K., Lazaridou M., Antonopoulos G., Makris J., Poliyiannakis J. (1996). Summary of the five principles suggested by Varotsos et al. [1996] and the additional questions raised in this debate. Geophys. Res. Lett..

[50] Varotsos P., Eftaxias K., Vallianatos F., Lazaridou M. (1996). Basic principles for evaluating an earthquake prediction method. Geophys. Res. Lett..

[51] Donges J.F., Donner R.V., Trauth M.H., Marwan N., Schellnhuber H.J., Kurths J. (2011). Nonlinear detection of paleoclimate-variability transitions possibly related to human evolution. Proc. Natl. Acad. Sci. USA.

[52] Siegmund J.F., Siegmund N., Donner R.V. (2017). CoinCalc—A new R package for quantifying simultaneities of event series. Comput. Geosci..

[53] Schleussner C.F., Donges J.F., Donner R.V., Schellnhuber H.J. (2016). Armed-conflict risks enhanced by climate-related disasters in ethnically fractionalized countries. Proc. Natl. Acad. Sci. USA.

[54] R Core Team (2013). R: A Language and Environment for Statistical Computing.

[55] Båth M. (1965). Lateral inhomogeneities of the upper mantle. Tectonophysics.

[56] Lombardi A.M. (2002). Probabilistic interpretation of Båth’s Law. Ann. Geophys..

[57] Console R., Lombardi A.M., Murru M., Rhoades D. (2003). Båth’s law and the self-similarity of earthquakes. J. Geophys. Res. Solid Earth.

[58] Papadopoulou K.A., Skordas E.S., Sarlis N.V. (2016). A tentative model for the explanation of Båth law using the order parameter of seismicity in natural time. Earthquake Sci..

[59] Peng H., Katsumi H., Maiko H., Jiancang Z., Chieh-Hung C., Febty F., Hiroki Y., Chie Y., Jann-Yenq L., Shuji Y. (2014). Statistical analysis of ULF seismomagnetic phenomena at Kakioka, Japan, during 2001–2010. J. Geophys. Res. Space Phys..

[60] Garber A., Hallerberg S., Kantz H. (2009). Predicting extreme avalanches in self-organized critical sandpiles. Phys. Rev. E.

[61] Sarlis N.V., Skordas E.S., Varotsos P.A. (2010). Order parameter fluctuations of seismicity in natural time before and after mainshocks. EPL.

[62] Caruso F., Kantz H. (2011). Prediction of extreme events in the OFC model on a small world network. Eur. Phys. J. B.

[63] Sarlis N., Skordas E., Varotsos P. (2011). The change of the entropy in natural time under time-reversal in the Olami-Feder-Christensen earthquake model. Tectonophysics.

[64] Sarlis N.V., Christopoulos S.R.G. (2012). Predictability of the coherent-noise model and its applications. Phys. Rev. E.

[65] Varotsos P.A., Sarlis N.V., Skordas E.S. (2012). Order parameter fluctuations in natural time and b-value variation before large earthquakes. Nat. Hazards Earth Syst. Sci..

[66] Sarlis N.V., Christopoulos S.R.G. (2014). Visualization of the significance of Receiver Operating Characteristics based on confidence ellipses. Comput. Phys. Commun..

[67] Christopoulos S.R.G., Sarlis N.V. (2017). An Application of the Coherent Noise Model for the Prediction of Aftershock Magnitude Time Series. Complexity.

[68] Mason S.J., Graham N.E. (2002). Areas beneath the relative operating characteristics (ROC) and relative operating levels (ROL) curves: Statistical significance and interpretation. Q. J. R. Meteorol. Soc..

[69] Dologlou E. (1993). A three year continuous sample of officially documented predictions issued in Greece using the van method: 1987–1989. Tectonophysics.

[70] Sarlis N.V., Skordas E.S., Lazaridou M.S., Varotsos P.A. (2008). Investigation of seismicity after the initiation of a Seismic Electric Signal activity until the main shock. Proc. Jpn. Acad. Ser. B Phys. Biol. Sci..

[71] Lazaridou-Varotsos M.S. (2013). Earthquake Prediction by Seismic Electric Signals. The Success of the VAN Method over Thirty Years.

[72] Varotsos P.A., Sarlis N.V., Skordas E.S., Tanaka H.K., Lazaridou M.S. (2006). Entropy of seismic electric signals: Analysis in the natural time under time reversal. Phys. Rev. E.

[73] Hayakawa M., Schekotov A., Potirakis S., Eftaxias K. (2015). Criticality features in ULF magnetic fields prior to the 2011 Tohoku earthquake. Proc. Jpn Acad. Ser. B Phys. Biol. Sci..

